# A Primary Retroperitoneal Mucinous Tumor

**DOI:** 10.1155/2015/157613

**Published:** 2015-03-19

**Authors:** Alicia A. Heelan Gladden, Max Wohlauer, Martine C. McManus, Csaba Gajdos

**Affiliations:** ^1^Department of Surgery, University of Colorado Denver, 12631 East 17th Avenue, C-305, Aurora, CO 80045, USA; ^2^Department of Pathology, University of Colorado Denver, Building L-15, Room 22xx, 12631 East 17th Avenue, Aurora, CO 80045, USA

## Abstract

A twenty-five-year-old female presented with a large retroperitoneal mass. Workup included history and physical exam, imaging, biopsy, colonoscopy, and gynecologic exam. After surgical resection, the mass was determined to be a primary retroperitoneal mucinous tumor (PRMT). Clinically and histologically, these tumors are similar pancreatic and ovarian mucinous neoplasms. PRMTs are rare and few case reports have been published. PRMTs are divided into mucinous cystadenomas, mucinous borderline tumors of low malignant potential, and mucinous carcinoma. These tumors have malignant potential so resection is indicated and in some cases adjuvant chemotherapy and/or surveillance imaging.

## 1. Introduction

This is the case of a twenty-five-year-old female who presented with a very large retroperitoneal mass, which was eventually diagnosed as a primary retroperitoneal mucinous tumor. Because this is a very rare tumor, few case reports exist describing this pathology. These tumors can be easily confused with more common tumors of pancreatic and ovarian origin. This case report provides a reminder that primary retroperitoneal mucinous tumors should be included in the differential diagnosis of retroperitoneal masses.

## 2. Case Report

**Figure 1:**
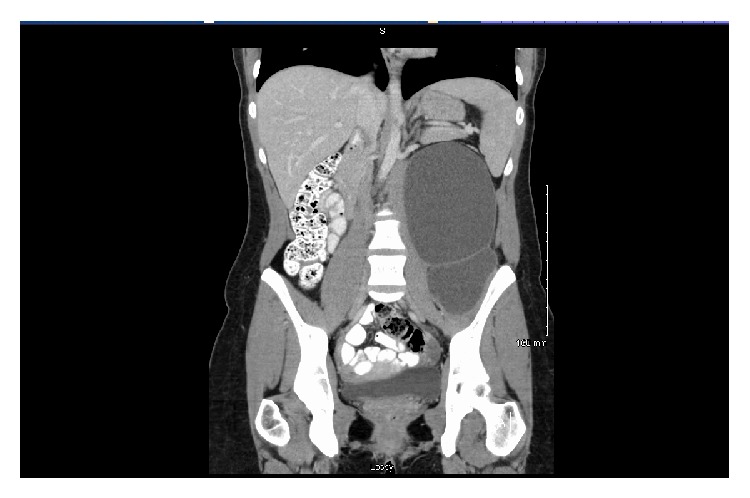
Computed tomographic scan showing a large retroperitoneal mass with cystic and solid components.

A twenty-five-year-old female presented to her student health clinic with increasing abdominal fullness, left-sided lower back pain, and left lateral thigh numbness that had been worsening over the course of several months. Family and medical history were unremarkable. Physical exam was significant for a large fixed mass in the left upper and lower quadrants of the abdomen. She had an elevated carcinoembryonic antigen of 49. Computed tomographic scan (CT) showed a large retroperitoneal mass with cystic and solid components (Figure 1).

The patient underwent ultrasound-guided biopsy of the solid portion of the mass. The specimen (evaluated by two pathologists) was consistent with well-differentiated adenocarcinoma, with intestinal morphology.

The patient subsequently underwent a colonoscopy and a gynecologic exam, neither of which revealed any abnormalities. Positron emission tomography scan showed avidity of the primary lesion but no distant metastatic disease.

**Figure 2:**
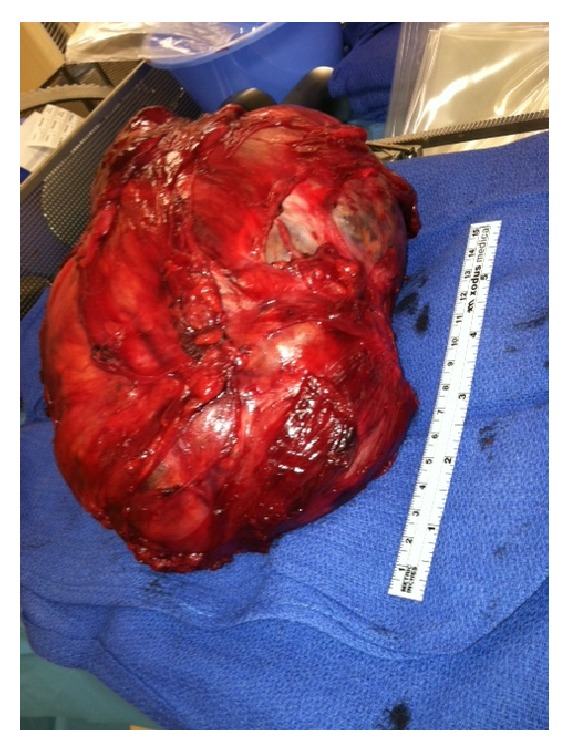
The gross specimen of a well-encapsulated 21 × 15 × 13 cm retroperitoneal mass.

A referral to our center was made. The patient underwent a laparotomy and resection of a well-encapsulated 21 × 15 × 13 cm retroperitoneal mass (Figure 2). The tumor was densely adherent to the iliac bone. Biopsies of the mesentery and the omentum were also taken. Her postoperative course was unremarkable and the patient was discharged on postoperative day number eight.

**Figure 3:**
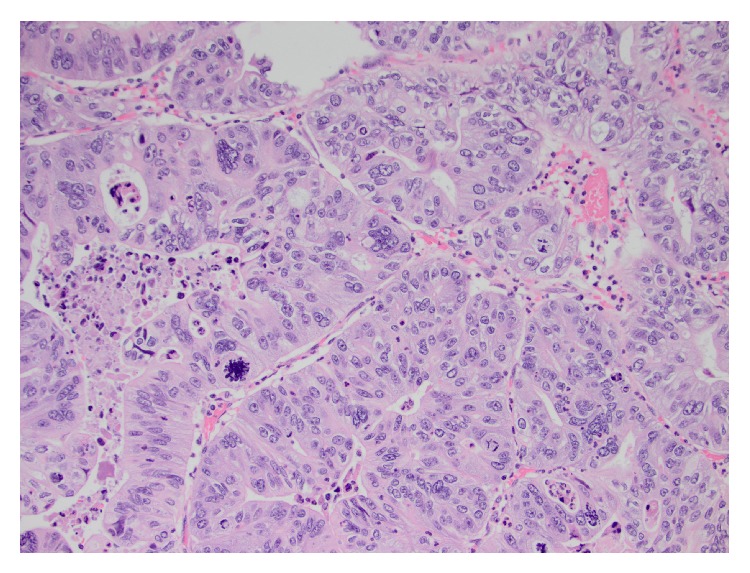
High power image of the tumor with multinucleated tumor cells and atypical mitotic figure. Image provided by Martine C McManus, MD.

The mass consisted of a cystic cavity lined by complex tubulopapillary formations composed of columnar cells with high-grade nuclei, multinucleated cells, and frequent atypical mitotic figures. Pathology determined that the mass was most consistent with a primary retroperitoneal mucinous tumor (PRMT) with an associated invasive moderate-to-poorly differentiated adenocarcinoma (Figure 3).

## 3. Discussion

Primary retroperitoneal mucinous tumors are extremely rare with only a few cases reported in the literature. These tumors usually present in premenopausal women, though it has also been described in postmenopausal females and men [1]. The chief complaint is often that of an asymptomatic abdominal mass, but it may also present with a vague abdominal complaint such as discomfort, pain, or fullness [2]. Workup should include imaging such as computed tomography (CT) scan. The CT usually reveals a cystic mass in the retroperitoneum with no associated lesions in nearby organs. Tissue diagnosis via biopsy is unlikely to be helpful in diagnosing a PRMT but may be helpful in ruling out other pathologies. It is recommended that all PRMTs be treated with resection, as these tumors do have malignant potential.

Histologically, PRMTs are divided into mucinous cystadenomas (MC), mucinous borderline tumors of low malignant potential (MLMP), and mucinous carcinomas (MCa). In a recent case series, half of the MLMP and MCa cases revealed goblet cells, consistent with intestinal differentiation and ovarian stroma [1]. This histologic pattern was found in our patient's mass making it difficult to differentiate between a PRMT and tumors of ovarian or pancreatic origin.

There are a number of  PRMT case reports in the literature and few report outcomes. Prognosis seems to correlate with histologic grading of the disease with MC tumors having a better prognosis than MCa tumors. One case series provided follow-up results for sixteen of eighteen cases. Follow-up ranged from 1 to 148 months. Of the sixteen, two patients died from the disease, and both had MCa with anaplastic carcinoma or sarcoma. The fourteen remaining patients were alive and only three of them had known disease at the time of follow-up [1].

It should be noted that consideration was given to the diagnosis of a pancreatic type mucinous cystic neoplasm (MCN) in our patient's case. It was postulated that the tumor may have originated from ectopic pancreas tissue or arose in the tail of the pancreas and then separated. However, there was no normal pancreatic tissue in the specimen nor were there any obvious abnormalities of the pancreas during surgery.

Pancreatic mucinous cystic neoplasms (MCN) are defined by the WHO as “cystic epithelial neoplasms that occur almost exclusively in women; do not communicate with the pancreatic ductal system; and are composed of columnar, mucin-producing epithelium, supported by ovarian-type stroma [3].” As alluded to earlier, PRMTs share histological similarities to ovarian mucinous cystadenomas and pancreatic mucinous neoplasms. In the presence of normal pancreas and ovaries, PRMT should be included in the differential diagnosis.

Chemotherapy and radiation therapy can be considered in those patients whose tumors display malignant components, as was the case with our patient. Follow-up should consist of surveillance imaging unless the tumor is found to be benign, as PRMTs have been reported to recur both locally and distally. Our patient received four months of cisplatin and gemcitabine. One and a half months after initiation of chemotherapy, our patient was found to have an iliac bone metastasis. This was treated with stereotactic radiation therapy in addition to the adjuvant chemotherapy. She currently has no evidence of disease and is undergoing surveillance imaging.
